# Mitochondrial genome sequencing of marine leukaemias reveals cancer contagion between clam species in the Seas of Southern Europe

**DOI:** 10.7554/eLife.66946

**Published:** 2022-01-18

**Authors:** Daniel Garcia-Souto, Alicia L Bruzos, Seila Diaz, Sara Rocha, Ana Pequeño-Valtierra, Camila F Roman-Lewis, Juana Alonso, Rosana Rodriguez, Damian Costas, Jorge Rodriguez-Castro, Antonio Villanueva, Luis Silva, Jose Maria Valencia, Giovanni Annona, Andrea Tarallo, Fernando Ricardo, Ana Bratoš Cetinić, David Posada, Juan Jose Pasantes, Jose MC Tubio

**Affiliations:** 1 Genomes and Disease, Centre for Research in Molecular Medicine and Chronic Diseases (CIMUS), Universidade de Santiago de Compostela Santiago de Compostela Spain; 2 Department of Zoology, Genetics and Physical Anthropology, Universidade de Santiago de Compostela Santiago de Compostela Spain; 3 Cancer Ageing and Somatic Mutation Programme, Wellcome Sanger Institute Cambridge United Kingdom; 4 Phylogenomics Lab, Universidade de Vigo Vigo Spain; 5 CINBIO, Universidade de Vigo Vigo Spain; 6 Galicia Sur Health Research Institute (IIS Galicia Sur), SERGAS-UVIGO Vigo Spain; 7 Centro de Investigación Mariña, Universidade de Vigo, ECIMAT Vigo Spain; 8 Instituto Español de Oceanografía (IEO), Centro Oceanográfico de Cádiz Cádiz Spain; 9 Laboratori d’Investigacions Marines i Aqüicultura, (LIMIA) - Govern de les Illes Balears Port d'Andratx, Balearic Islands Spain; 10 Instituto de Investigaciones Agroambientales y de Economía del Agua (INAGEA) (INIA-CAIB-UIB) Palma de Mallorca, Balearic Islands Spain; 11 Stazione Zoologica Anton Dohrn Napoli Italy; 12 ECOMARE, Centre for Environmental and Marine Studies (CESAM), Department of Biology, University of Aveiro, Santiago University Campus Aveiro Portugal; 13 Department of Aquaculture, University of Dubrovnik Dubrovnik Croatia; 14 Department of Biochemistry, Genetics and Immunology, Universidade de Vigo Vigo Spain; 15 Centro de Investigación Mariña, Universidade de Vigo Vigo Spain; Pacific Northwest Research Institute United States; Max Planck Institute for Developmental Biology Germany

**Keywords:** transmissible cancer, cancer genomes, marine leukaemias, Other

## Abstract

Clonally transmissible cancers are tumour lineages that are transmitted between individuals via the transfer of living cancer cells. In marine bivalves, leukaemia-like transmissible cancers, called hemic neoplasia (HN), have demonstrated the ability to infect individuals from different species. We performed whole-genome sequencing in eight warty venus clams that were diagnosed with HN, from two sampling points located more than 1000 nautical miles away in the Atlantic Ocean and the Mediterranean Sea Coasts of Spain. Mitochondrial genome sequencing analysis from neoplastic animals revealed the coexistence of haplotypes from two different clam species. Phylogenies estimated from mitochondrial and nuclear markers confirmed this leukaemia originated in striped venus clams and later transmitted to clams of the species warty venus, in which it survives as a contagious cancer. The analysis of mitochondrial and nuclear gene sequences supports all studied tumours belong to a single neoplastic lineage that spreads in the Seas of Southern Europe.

## eLife digest

In humans and other animals, cancer cells divide excessively, forming tumours or flooding the blood, but they rarely spread to other individuals. However, some animals, including dogs, Tasmanian devils and bivalve molluscs like clams, cockles and mussels, can develop cancers that are transmitted from one individual to another. Despite these cancers being contagious, each one originates in a single animal, meaning that even when the cancer has spread to many individuals, its origins can be traced through its DNA.

Cancer contagion is rare, but transmissible cancers seem to be particularly common in the oceans. In fact, 7 types of contagious cancer have been described in bivalve species so far. These cancers are known as ‘hemic neoplasias’, and are characterized by the uncontrolled division of blood-like cells, which can be released by the host they developed in, and survive in ocean water. When these cells encounter individuals from the same species, they can infect them, causing them to develop hemic neoplasia too

There are still many unanswered questions about contagious cancers in bivalves. For example, how many species do the cancers affect, and which species do the cancers originate in? To address these questions, Garcia-Souto, Bruzos, Díaz et al. gathered over 400 specimens of a species of clam called the warty venus clam from the coastlines of Europe and examined them for signs of cancer. Clams collected in two regions of Spain showed signs of hemic neoplasia: one of the populations was from the Balearic Islands in the Mediterranean Sea, while the other came from the Atlantic coast of northwestern Spain.

Analyzing the genomes of the tumours from each population showed that the cancer cells from both regions had likely originated in the same animal, indicating that the cancer is contagious and had spread through different populations. The analysis also revealed that the cancer did not originally develop in warty venus clams: the cancer cells contained DNA from both warty venus clams and another species called striped venus clams. These two species live close together in the Mediterranean Sea, suggesting that the cancer started in a striped venus clam and then spread to a warty venus clam. To determine whether the cancer still affected both species, Garcia-Souto, Bruzos, Díaz et al. screened 200 striped venus clams from the same areas, but no signs of cancer were found in these clams. This suggests that currently the cancer only affects the warty venus clam.

These findings confirm that contagious cancers can jump between clam species, which could be threat to the marine environment. The fact that the cancer was so similar in clams from the Atlantic coast and from the Mediterranean Sea, however, suggests that it may have emerged very recently, or that human activity helped it to spread from one place to another. If the latter is the case, it may be possible to prevent further spread of these sea-borne cancers through human intervention.

## Introduction

Cancers are clonal cell lineages that arise due to somatic changes that promote cell proliferation and survival (Stratton et al., 2009). Although natural selection operating on cancers favours the outgrowth of malignant clones with replicative immortality, the continued survival of a cancer is generally restricted by the lifespan of its host. However, clonally transmissible cancers – from now on, transmissible cancers – are somatic cell lineages that are transmitted between individuals via the transfer of living cancer cells, meaning that they can survive beyond the death of their hosts (Murchison, 2008). Naturally occurring transmissible cancers have been identified in dogs (Murgia et al., 2006; Murchison et al., 2014; Baez-Ortega et al., 2019), Tasmanian devils (Murchison et al., 2012; Pye et al., 2016) and, more recently, in marine bivalves (Metzger et al., 2015; Metzger et al., 2016; Yonemitsu et al., 2019).

Hemic neoplasia (HN), also called disseminated neoplasia, is a type of leukaemia cancer found in multiple species of bivalves, including oysters, mussels, cockles, and clams (Carballal et al., 2015). Although these leukaemias represent different diseases across bivalve species, they have been classically grouped under the same term because neoplastic cells share morphological features (Carballal et al., 2015). Some HNs have been proven to have a clonal transmissible behaviour (Metzger et al., 2015), in which neoplastic cells, most likely haemocytes (i.e. the cells that populate the haemolymph and play a role in the immune response), are likely to be transmitted through marine water. In late stages of the disease, leukaemic cells invade the surrounding tissues and, generally, animals die because of the infection (Carballal et al., 2015), although remissions have also been described (Burioli et al., 2019). Despite the observation that leukaemic cells are typically transmitted between individuals from the same species, on occasion they can infect and propagate across populations from a second, different bivalve species (Metzger et al., 2016; Yonemitsu et al., 2019). Hence, these cancers represent a potential threat for the ecology of the marine environment, which argues for the necessity of their identification and characterization for their monitoring and prevention.

Here, we use multiplatform next-generation genome sequencing technologies, including Illumina short reads and Oxford Nanopore long reads, together with cytogenetics, electron microscopy, and cytohistological approaches to identify, characterize, and decipher the evolutionary origin of a new marine leukaemia that is transmitted between two different clam species that inhabit the Seas of Southern Europe, namely warty venus (*Venus verrucosa*) and striped venus (*Chamelea gallina*) (Video 1).

**Video 1.** Mitochondrial genome sequencing of marine leukaemias reveals cancer contagion between clam species in the Seas of Southern Europe. Infographic video outlining the main findings of the research carried out.

## Results and discussion

**Figure 1.**
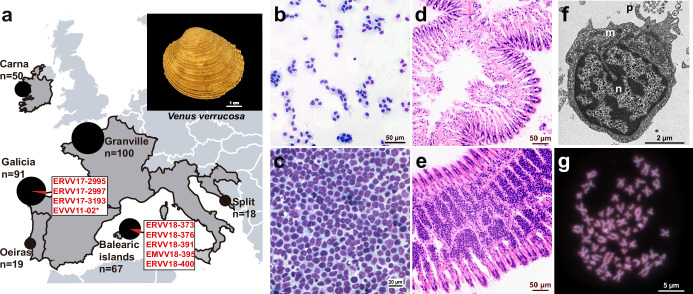
Geographical location of warty venus (*V.* *verrucosa*) specimens and diagnosis of hemic neoplasia. (**a**) Locations of *V. verrucosa* clams collected for this study and specimens diagnosed with hemic neoplasia. Size of the pie charts correlates with the number of samples collected (number of samples ‘*n*’ is shown together with each pie chart). Pie charts show the proportion of samples with hemic neoplasia (black, no neoplastic specimens; red, neoplastic specimens). Codes of neoplastic samples are shown. Top-right corner shows a representative specimen of the species *V. verrucosa*. (**b**) Cytological examination of haemolymph smear (Hemacolor stain) from a healthy (N0) specimen, ERVV17-2963, shows normal haemocytes. (**c**) Haemolymph smear of a *V. verrucosa* specimen with high-grade (N3 stage) hemic neoplasia, ERVV17-3193, shows neoplastic cells that replaced normal haemocytes. (**d**) Detail of haematoxylin and eosin-stained of histological section from the gills of the healthy (N0) specimen ERVV17-2990. (**e**) Same for ERVV17-2995, a specimen infected with a high-grade (N3 stage) hemic neoplasia, showing neoplastic cells infiltrating the gills. (**f**) Transmission electron microscopy analysis of a *V. verrucosa* hemic neoplasia tumour cell shows a round shape, pseudopodia ‘p’, pleomorphic nucleus ‘n’ with scattered heterochromatin, and mitochondria ‘m’. (**g**) Metaphase chromosomes from a neoplastic cell found in the gills of the *V. verrucosa* specimen EVVV11-02, showing abnormal chromosome number (>19 pairs) and abnormal chromosome morphology. Chromosomes stained with 4′,6-DiAmidino-2-PhenylIndole (DAPI) and Propidium Iodide (PI).

We investigated the prevalence of HN in the warty venus clam (*V. verrucosa*), a saltwater bivalve found in the Atlantic Coast of Europe and the Mediterranean Sea. We collected 345 clam specimens from six sampling regions in the Atlantic and the Mediterranean coasts of Europe across five different countries, including Spain, Portugal, France, Ireland, and Croatia (Figure 1a; Supplementary file 1). Cytohistological examination identified HN-like tumours in eight specimens from two sampling points in Spain (Figure 1b–e; Figure 1—figure supplement 1). Three HN-positive specimens (ERVV17-2995, ERVV17-2997, and ERVV17-3193) were collected in Galicia, northwest of the Iberian Peninsula in the Atlantic Ocean, and another five specimens (EMVV18-373, EMVV18-376, EMVV18-391, EMVV18-395, and EMVV18-400) were collected in the Balearic Islands, bathed by the Mediterranean Sea (Figure 1a; Supplementary file 1). Four of these specimens (ERVV17-2995, ERVV17-3193, EMVV18-391, and EMVV18-395) showed a severe form of the disease – classified as N3 stage – which is characterized by high levels of neoplastic cells infiltrating the gills, different levels of infiltration of the digestive gland and gonad, and low/very low infiltration of the mantle and foot (Figure 1d,e; Figure 1—figure supplement 1); one specimen (EMVV18-400) was found that was affected with an intermediate form of the disease – N2 stage – characterized by low levels of neoplastic cells infiltrating the gill vessels, digestive gland, and gonad, but not the foot (Figure 1—figure supplement 1); and three specimens (ERVV17-2997, EMVV18-373, and EMVV18-376) were diagnosed with a light form of the disease – N1 stage – characterized by low levels of neoplastic cells infiltrating the gills vessels only, and no infiltration in the remaining tissues (Figure 1—figure supplement 1). Electron microscopy analysis through gill’s ultrathin sections from two neoplastic warty venus specimens (ERVV17-2995 and ERVV17-3193) revealed tumour cells with a round shape and a pleomorphic nucleus, which are morphological features that generally characterize bivalves’ HN (Figure 1f; Figure 1—figure supplement 2). Finally, one additional neoplastic warty venus specimen (EVVV11-02) was included in the study. The animal, which was sampled in 2011 in Galicia and came from a private collection, showed abnormal metaphases in the gills that were suggestive of HN. Although the species typically shows a 2n = 38 karyotype with metacentric chromosomes that are homogeneous in size (García-Souto et al., 2015), the tumoural metaphases from this individual showed around 100 chromosomes that were variable in size and shape (Figure 1g).

**Figure 1—figure supplement 1.**
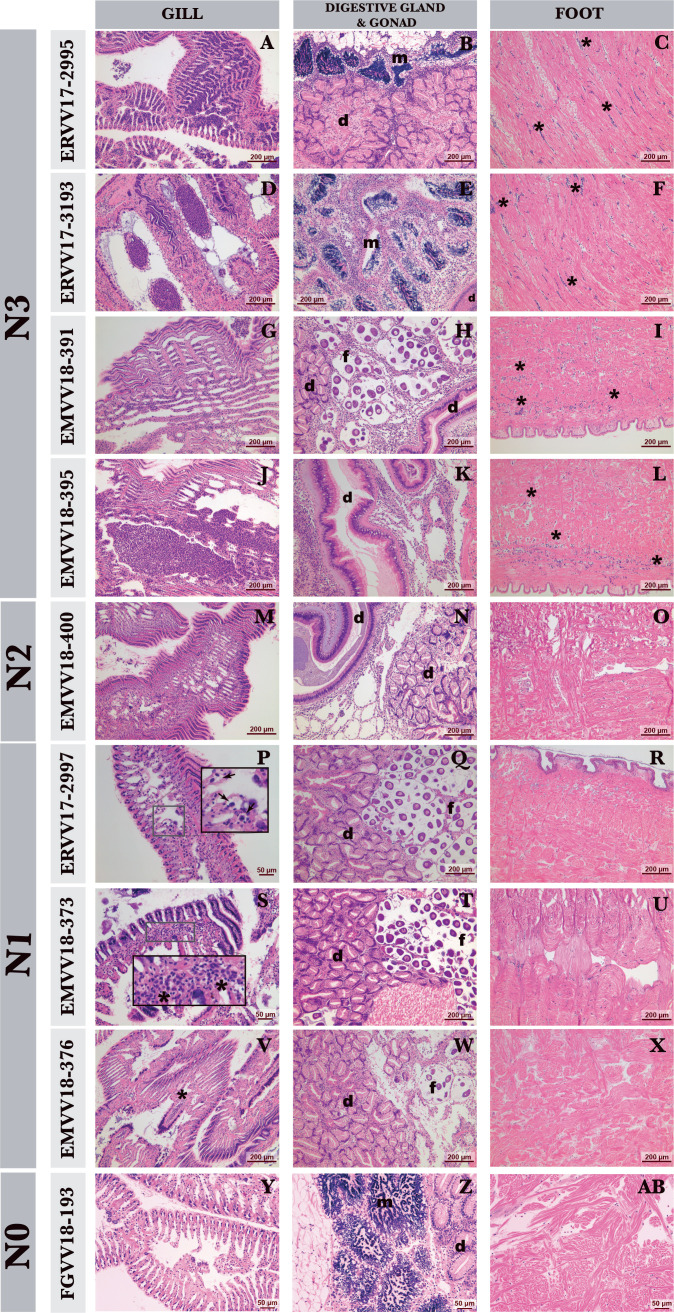
Histological diagnosis of hemic neoplasia in warty venus (*V. verrucosa*) specimens. Haematoxylin and eosin-stained photomicrographs of gill, digestive (d), gonad (male [m] and female [f]), and foot of warty venus (*V. verrucosa*) specimens diagnosed with different stages of hemic neoplasia: high (N3), medium (N2), light (N1), and healthy (N0). In the N3 stage, neoplastic cells infiltrate the connective tissue and vessels of different organs (**A–L**), and show low infiltration of foot (**C, F, I, L**). In N2 stage, cell groups are observed in different organs such as gills (**M**) or gonad and digestive gland (N) and are not detected in the foot (**O**). In N1 stage, groups of neoplastic or isolated cells are detected in gill sinuses (**P, S, V**) and not found in either gonad and digestive gland (Q, T, W) or foot (R, U, X). N0 stage is completely devoid of any trace of hemic neoplasia at either gill, digestive gland, and gonad and foot (**Y, Z, AB**). Arrows show isolated cells. Asterisks show groups of neoplastic cells. Scale bar 200 µm.

**Figure 1—figure supplement 2.**
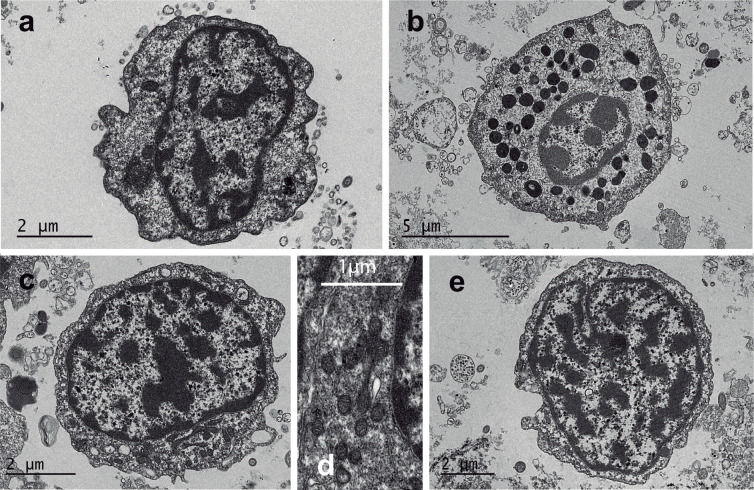
Transmission electron microscopy (TEM) of healthy and neoplastic *V. verrucosa* specimens. TEM cellular ultrastructure of healthy warty venus haemocytes, hyalinocyte (a), and granulocyte (b) and neoplastic cells (**c, d, **e). (d) shows mitochondria of neoplastic cells in detail. Processed samples: ERVV17-2992 (**a**), ERVV17-2993 (**b**), ERVV17-2995 (**c, , **), and ERVV17-3193 (**d, **e).

**Figure 2.**
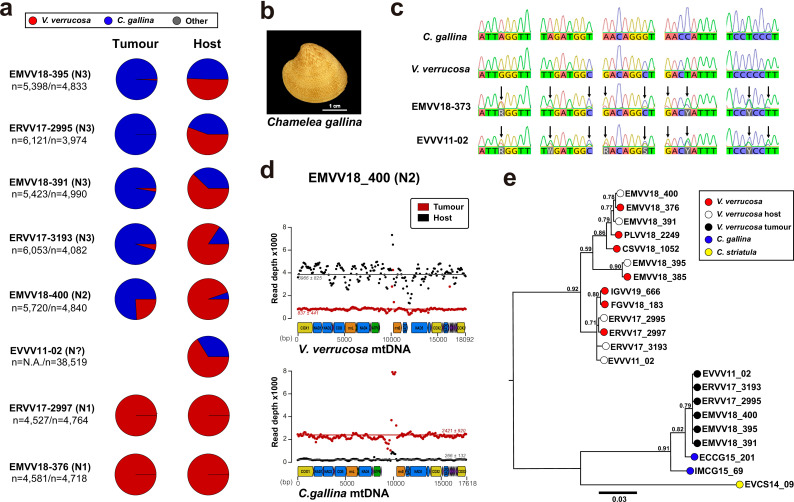
Mitochondrial DNA sequencing and phylogenetic analyses reveal cancer contagion between warty venus (*V.* *verrucosa*) and striped venus (*C. gallina*) clam species. (**a**) In eight warty venus specimens sequenced with Illumina paired-ends, the pie charts show the proportion of reads mapping *Cox1* reference sequences from 137 different *Verenidae* species, including *V. verrucosa* (red), *C. gallina* (blue), and the remaining species (grey). Two different tissues were sequenced: the tumour tissue (left pie chart), typically haemolymph, and the host/matched-normal tissue (right pie chart), typically foot. Note that for specimen EVVV11-02 only the host/matched-normal tissue (gills) was available. ‘*n*’ denotes the total number of reads mapping the *Cox1* reference for the tumour tissue (left), and the host tissue (right). (**b**) Representative specimen of the species *C. gallina*. (**c**) Capillary sequencing electropherograms of mitochondrial *Cox1* gene fragments from two neoplastic *V. verrucosa* specimens (EMVV18-373 and EVVV11-02) and two healthy reference specimens from *V. verrucosa* and *C. gallina*. The results show overlapping peaks (arrows) in the sequenced tissues from the neoplastic animals, which suggest coexistence of mitochondrial DNA (mtDNA) haplotypes from two clam species. (**d**) In *V. verrucosa* neoplastic (N2-stage) specimen EMVV18-400, mtDNA read depth shows different proportion of warty venus and striped venus mtDNA haplotypes in the tumour tissue (haemolymph) and the matched-normal tissue (foot). (**e**) Molecular phylogeny using Bayesian inference inferred on the alignment of all mitochondria coding genes and rRNA gene sequences (15 loci) that includes six neoplastic *V. verrucosa* specimens with evidence of cancer contagion from *C. gallina*. Bootstrap values are shown above the branches.

To obtain some biological insights into the clonal dynamics of this cancer, we carried out whole-genome sequencing with Illumina paired-ends in DNA samples isolated from the tumoural haemolymph from eight out of nine neoplastic specimens mentioned above (Table 1). Their feet were also sequenced, as foot typically represents the tissue with lower infiltration of neoplastic cells, making it a good candidate tissue to act as ‘matched-normal’ (i.e. host tissue). As for the animal with an abnormal karyotype (EVVV11-02) that was compatible with HN, we sequenced the only tissue available, which were gills (Table 1). Only one neoplastic specimen (EMVV18-373) that had a very low proportion of tumour cells in its haemolymph was excluded from the sequencing. Then, we mapped the paired-end reads onto a dataset containing non-redundant mitochondrial Cytochrome C Oxidase subunit 1 (*Cox1*) gene references from 118 Venerid clam species. In six out of eight sequenced neoplastic specimens, the results revealed an overrepresentation (>99%) of reads in the sequenced tissues mapping to *Cox1* DNA sequences that exclusively identified two different clam species (Figure 2a): the expected one, warty venus clam (*V. verrucosa*), and a second, unexpected one, the striped venus (*C. gallina*), a clam that inhabits the Mediterranean Sea (Figure 2b). Preliminary analysis by PCR and capillary sequencing of *Cox1* in the haemolymph of two neoplastic specimens, EMVV18-373 and EVVV11-02, revealed an electropherogram with overlapping peaks apparently containing two different haplotypes that match the reference *Cox1* sequences for warty and striped venus (Figure 2c).

**Figure 2—figure supplement 1.**
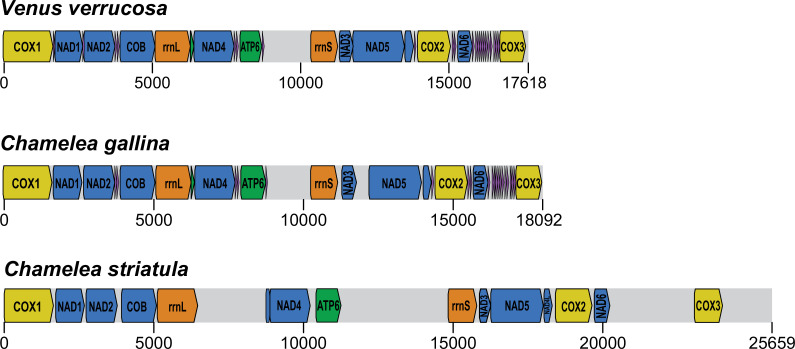
Draft reference mitochondrial DNA (mtDNA) genome assemblies reconstructed for *V. verrucosa*, *C. gallina*, and *C. striatula*. Preliminary ‘reference’ mtDNA genome assembly obtained from paired-end sequencing data, together with gene annotation.

**Figure 2—figure supplement 2.**
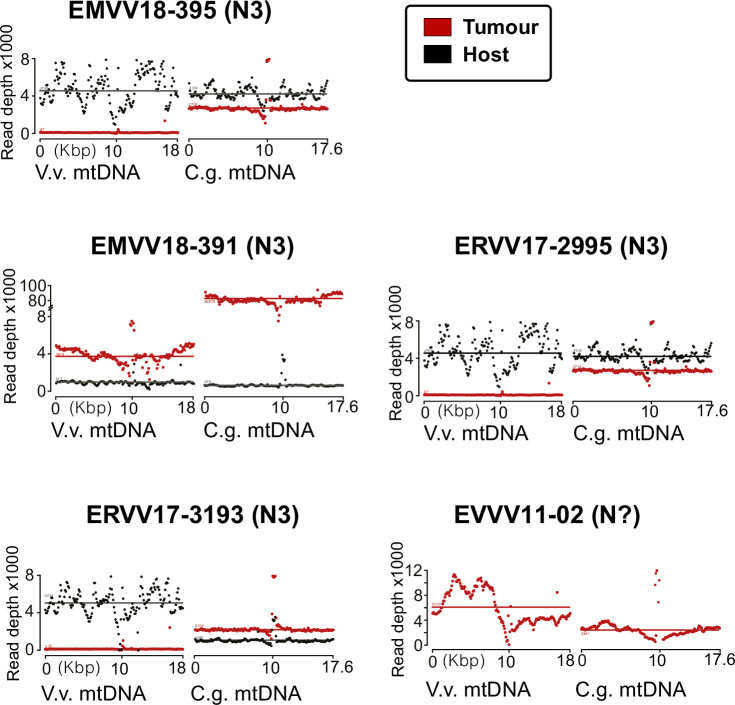
Read depth analysis of the mitochondrial DNA (mtDNA) confirms coexistence of two different clam species haplotypes. mtDNA read depth shows different proportion of warty venus and striped venus mtDNA haplotypes in the tumour tissue (haemolymph) and the matched-normal tissue (foot) in the remaining five tumours analysed.

**Table 1.** Clam specimens and tissues sequenced with Illumina paired-ends. Sixteen specimens (eight neoplastic and eight non-neoplastic) from three different clam species (*V. verrucosa*, *C. gallina*, and *C. striatula*) were sequenced with Illumina paired-ends. Columns 5 and 6 show the number of reads generated for the host tissue (when neoplastic, matched-normal tissue was foot) and the tumoural haemolymph, respectively. (*) denotes the only available tissue from this neoplastic animal, collected in 2011, were gills. (#) denotes hemic neoplasia stage was not determined because cytohistological examination was not possible in this individual, which was diagnosed by cytogenetics.

Clam species	Specimen origin	Specimen code	Diagnosis	Foot reads	Haemolymph reads
*V. verrucosa*	Galicia, Spain	ERVV17-2995	N3	833 M	919 M
*V. verrucosa*	Galicia, Spain	ERVV17-2997	N1	766 M	598 M
*V. verrucosa*	Galicia, Spain	ERVV17-3193	N3	739 M	850 M
*V. verrucosa*	Balearic Islands, Spain	EMVV18-376	N1	784 M	849 M
*V. verrucosa*	Balearic Islands, Spain	EMVV18-391	N3	617 M	623 M
*V. verrucosa*	Balearic Islands, Spain	EMVV18-395	N3	697 M	679 M
*V. verrucosa*	Balearic Islands, Spain	EMVV18-400	N1	782 M	1133 M
*V. verrucosa*	Galicia, Spain	EVVV11-02	N^#^	743 M*	–*
*V. verrucosa*	Split, Croatia	CSVV18-1052	Healthy	161 M	–
*V. verrucosa*	Balearic Islands, Spain	EMVV18-385	Healthy	143 M	–
*V. verrucosa*	Granville, France	FGVV18-183	Healthy	752 M	–
*V. verrucosa*	Carna, Ireland	IGVV19-666	Healthy	155 M	–
*V. verrucosa*	Oeiras, Portugal	PLVV18-2249	Healthy	163 M	–
*C. gallina*	S.Benedetto, Italy	IMCG15-69	Healthy	147 M	–
*C. gallina*	Cadiz, Spain	ECCG15-201	Healthy	752 M	–
*C. striatula*	Galicia, Spain	EVCS14-09	Healthy	706 M	–

These results suggested cancer contagion between the two clam species of the family *Veneridae*. Hence, to decipher the origins of this clam neoplasia, we further analysed the mitochondrial DNA (mtDNA) from the two species involved and the tumours. Firstly, we performed multiplatform genome sequencing, including Illumina short reads and Oxford Nanopore long reads, on canonical individuals from the two species to obtain a preliminary assembly of the mitogenomes of *V. verrucosa* and *C. gallina*. These reconstructions resulted in 18,092- and 17,618-bp long mtDNA genomes for the warty venus and the striped venus clam, respectively (Figure 2—figure supplement 1). The comparative analysis of the nucleotide sequences from both mitogenomes confirms that, although both species are relatively close within the subfamily *Venerinae* (Canapa et al., 1996), they represent distinct sister species, showing a Kimura’s two-parameter nucleotide distance (K2P) equal to 21.13%. Then, we mapped the paired-end sequencing data from the six neoplastic specimens with evidence of interspecies cancer transmission onto the two reconstructed species-specific mtDNA genomes. This approach confirmed the coexistence of two different mtDNA haplotypes in the six examined neoplastic samples, matching the canonical mtDNA genomes from the two clam species. For example, in a N2-stage specimen (EMVV18-400), this analysis revealed different proportion of tumour and host mtDNA molecules in the two tissue types sequenced (Figure 2d). Here, the striped venus mtDNA results the most abundant in the haemolymph, in which tumour cells are dominant over the remaining cell types, and the lower in the matched-normal tissue (i.e. infiltrated foot), where tumour cells represent a minor fraction of the total. Similar results were obtained for the remaining five neoplastic individuals (Figure 2—figure supplement 2).

To further investigate the evolutionary origins and geographic spread of this cancer, we sequenced with Illumina paired-ends an additional set of eight healthy (i.e. non-neoplastic) clams from three different *Veneridae* species, including five more warty venus specimens (EMVV18-385, IGVV19-666, FGVV18-183, CSVV18-1052, and PLVV18-2249) from five different countries, two striped venus specimens (IMCG15-69 and ECCG15-201) from two countries, and one specimen (EVCS14-09) from its sibling species *Chamelea striatula*, a type of striped venus clam that inhabits the Atlantic Ocean from Norway to the Gulf of Cadiz in Spain. This made a total of 16 *Veneridae* specimens sequenced, all listed in Table 1 (see also Supplementary file 1). The complete mitochondrial genomes from all tumoural and healthy *V. verrucosa* specimens (13 individuals), 2 *C. gallina*, and 1 from its sibling species *C. striatula*, were individually de novo assembled from the sequencing reads. As expected, this approach reconstructed two different haplotypes in six out eight sequenced neoplastic animals, supporting the presence of mtDNA from two different species. Despite the high sequencing coverage obtained for these individuals (Table 1), we did not find foreign reads in the N1 tumours (ERVV17-2997 and EMVV18-373), most likely due to a low proportion of neoplastic cells in the haemolymph and the matched-normal tissue. Then, we performed a phylogenetic analysis based on the alignment of these mitochondrial genomes (13 coding and 2 RNA gene sequences, altogether encompassing ~14 kb). The results show that tumour and non-tumour sequences from neoplastic warty venus specimens define two well-differentiated clades, and that tumoural warty venus sequences are all identical and closer to striped venus mtDNA than to its own (warty venus) (Figure 2e). Overall, these data support the existence of a single cancer clone originated in the striped venus clam *C. gallina* that was transmitted to *V. verrucosa*.

**Figure 3.**
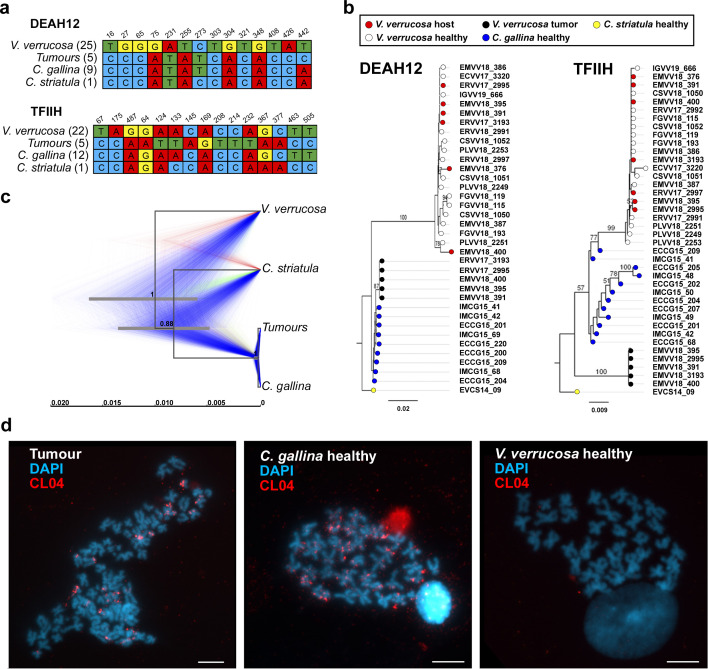
Nuclear DNA sequencing and phylogenetic analyses confirm a single cancer lineage spreading in populations of the warty venus (*V.* *verrucosa*) that originated in the striped venus (*C. gallina*). (**a**) Single-nucleotide variants discriminating between V. *verrucosa* tumours and the three canonical species (*V. verrucosa*, *C. gallina*, and *C. striatula*) along a 441- and a 559-bp long fragments of nuclear genes *DEAH12* and *TFIIH*, respectively. (**b**) Maximum Likelihood molecular phylogenies based on the two fragments of the nuclear DNA markers *DEAH12* and *TFIIH*. Bootstrap support values (500 replicates) from Maximum Likelihood analyses above 50 are shown on the corresponding branches. (**c**) Multispecies coalescent (MSC) tree of *V. verrucosa*, their tumours and *Chamelea* sp. based on the entire mitochondrial DNA (mtDNA) and the two nuclear markers, *DEAH12* and *TFIIH*. A maximum clade credibility (MCC) tree is shown, with posterior probabilities below the branches, and 95% highest probability density (HPD) intervals of node heights as grey bars. The trees distribution shown includes 1000 trees and represents the range of alternative topologies, in which blue is the most common set of topologies, red the second most common one, and green the remaining. (**d**) Fluorescence in situ hybridization (FISH) to specifically detect the satellite DNA CL4 in one *V. verrucosa* tumour and healthy specimens from the species *C. gallina* and *V. verrucosa* shows probes accumulate in heterochromatic regions, mainly in subcentromeric and subtelomeric positions, from the chromosomes of the tumour and the healthy *C. gallina* tested but not in healthy *V. verrucosa*.

Transmissible cancers are known to occasionally acquire mitochondria from transient hosts (Strakova et al., 2016; Strakova et al., 2020), which can lead to misinterpretation of their evolutionary history. Thus, we looked for nuclear markers to confirm the striped venus origin of this cancer lineage. We performed a preliminary draft assembly of the warty venus and the striped venus nuclear ‘reference’ genomes, using the paired-end sequencing data from two non-neoplastic animals. Then, we used bioinformatic approaches to find single copy nuclear genes that were homologous between the two species, identifying two confident candidate genomic regions (see Methods): a 2.9-kb long region from *DEAH12*, a gene that encodes for an ATP-dependent RNA helicase, and a 2.2-kb long fragment from the Transcription Factor II Human-like gene, *TFIIH*. With the idea of finding differentially fixed single-nucleotide variants (SNVs) between both species, we performed PCR amplification and capillary sequencing on a 441 bp fragment from the *DEAH12*, and a 559 bp fragment from *TFIIH*, in 2 cohorts of non-neoplastic warty venus specimens (12 for *DEAH12* and 15 for *TFIIH*), 2 cohorts of non-neoplastic striped venus (9 for *DEAH12* and 12 for *TFIIH*), and 1 specimen of its sister species *C. striatula*. This analysis provided 14 and 15 sites, respectively, for the *DEAH12* and the *TFIIH* loci, with fixed SNVs (allele frequency >95%) that allowed to discriminate between the 3 relevant species and the tumour (Figure 3a). These variants were employed to identify the Illumina reads from each sequenced warty venus neoplastic specimens that were specific for either warty venus or striped venus, which allowed to obtain the consensus sequences that corresponded to the tumour tissue and the non-affected tissue from each neoplastic individual. At the end of this process, we performed Maximum Likelihood phylogenetic reconstructions from these individual nuclear consensus sequences. On the one hand, the phylogeny for the *DEAH12* locus confirmed both the monophyly of the tumoural sequences and their closer relationship to *C. gallina* than to the host species (Figure 3b), which were also observed in the mtDNA analysis. However, the phylogeny derived from the *TFIIH* locus showed that, although the tumours remained monophyletic, they were positioned in a basal branch relative to *C. gallina* and *V. verrucosa* (Figure 3b). Hence, to resolve these differences we also obtained a multilocus species tree based on the alignment of both the mtDNA and the two nuclear genes. This new phylogeny confirmed that warty venus tumours are closer to striped venus specimens than to non-neoplastic warty venus sequences from the same diseased specimens, while the non-neoplastic sequences conformed a more distant warty venus lineage (Figure 3c).

**Figure 3—figure supplement 1.**
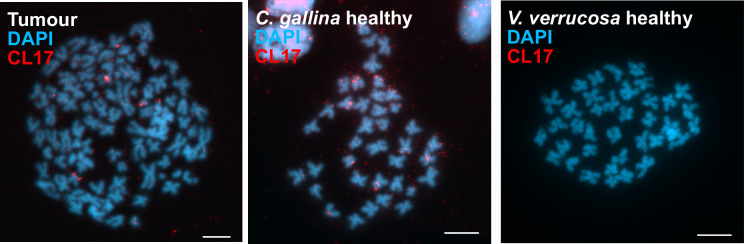
CL17 satellite DNA supports tumour transmission from *C. gallina*. Fluorescence in situ hybridization (FISH) to specifically detect the satellite DNA CL17 in one *V. verrucosa* tumour and healthy specimens from the species *C. gallina* and *V. verrucosa* shows probes accumulate in heterochromatic regions, mainly in subcentromeric and subtelomeric positions, from the chromosomes of the tumour and the healthy *C. gallina* tested but not in healthy *V. verrucosa*.

To obtain further evidence on the striped venus origin of this clam’s neoplasia, we performed a comparative screening of tandem repeats in the genomes of *C. gallina* and *V. verrucosa* using fluorescence in situ hybridization (FISH) (Figure 3d; Figure 3—figure supplement 1). We focused on two satellite DNA repeats, namely CL4 and CL17. The satellites represent repeats of 332- and 429-bp long monomers, respectively, and were identified in a preliminary bioinformatics screening of the striped venus reference genome (see methods). This FISH approach revealed that the mentioned repeats are very abundant in heterochromatic regions from the genomes of the canonical striped venus and the neoplastic warty venus specimens tested (Figure 3d; Figure 3—figure supplement 1). However, the repeats were absent in the metaphases from all the healthy warty venus individuals (Figure 3d; Figure 3—figure supplement 1). These results suggest that the relevant chromosomes with CL4 and CL17 satellites found in neoplastic warty venus specimens derive from *C. gallina*, supporting that a tumour originated in *C. gallina* was transmitted to *V. verrucosa*.

To find out whether this cancer is present in the clam species where it first arose, we performed a screening for its presence in natural populations of striped venus clams from the species *C. gallina* (*n* = 213) and *C. striatula* (*n* = 9) at five additional sampling points across two countries (Supplementary file 1), including Spain (*n* = 115) and Italy (*n* = 107). Histological analyses did not show any traces of HN in these specimens. The virtual absence of this tumour in natural populations of striped venus clams may suggest that today this leukaemia is being mainly, if not exclusively, transmitted between specimens of the recipient species, warty venus. However, further sampling in other regions across the striped venus area of distribution may be necessary to confirm these findings.

Overall, the results provided here reveal the existence of a transmissible leukaemia originated in a striped venus clam, most likely *C. gallina*, which was transmitted to a second species, the warty venus clam (*V. verrucosa*), and among whose specimens it currently propagates. We identified this parasitic cancer in warty venus clams from two sampling points that are more than 1000 nautical miles away in the coasts of Spain, bathed by two different seas, the Atlantic Ocean and the Mediterranean Sea. The analysis of mitochondrial and nuclear gene sequences revealed no nucleotide diversity within the seven tumours sequenced, which supports that all belong to the same neoplastic lineage that spreads between Veneridae clams in the Seas of Southern Europe. Although we ignore the age of this cancer clone, we can confirm it arose before 2011, when the neoplastic warty venus specimen EVVV11-02 was collected. The apparent lack of genetic variation between all tumours, even from distant sampling points, suggests either that this cancer is very recent, or that it may have been unintentionally scattered by the action of man, a way of transmission that has been proposed for other bivalve transmissible cancers (Yonemitsu et al., 2019).

## Materials and methods

### Sampling of clam specimens

We collected 570 clam specimens from three different species, from the following countries and locations (Supplementary file 1). *V. verrucosa* clams were collected in Spain (Galicia, *n* = 90; Balearic Islands, *n* = 67), France (Granville, *n* = 100), Croatia (Split, *n* = 18), Portugal (Oeiras, *n* = 19), and Ireland (Carna, *n* = 50). *C. gallina* clams were collected in Spain (Cadiz, *n* = 50; Mallorca, *n* = 50) and Italy (Naples, *n* = 50; Cattolica, *n* = 57). *C. striatula* clams were collected in Spain (Combarro, *n* = 9). Additionally, we recruited samples from the following specimens from private collections: one *V. verrucosa* clam collected in 2011 in Spain (Islas Cies), four *C. gallina* collected in 2015 in Italy (San Benedetto de Tronto), five *C. gallina* collected in 2015 in Spain (Huelva), and one *C. striatula* collected in 2014 in Spain (Marin).

### Diagnosis of HN

We followed standard cytological and/or histological protocols to test and diagnose HN in the clam specimens. However, only histological examination resulted decisive for the diagnosis, particularly in early stages of the disease. Briefly, for each animal, we extracted 300–2000 ml of haemolymph from the posterior adductor muscle using a 5 ml syringe with a 23 G needle. The haemolymph (50 ml) was diluted in cold Alserver’s antiaggregant solution to a 1:4 concentration, and spotted by centrifugation (130 × *g*, 4°C, 7 min) onto a microscope slide using cytology funnel sample chambers to produce a cell monolayer. Haemolymph smears were fixed and stained with Hemacolor solutions from Sigma-Aldrich and subsequently examined with a light microscope for the diagnosis of HN. Tissues (visceral mass, gills, mantle, and foot) were dissected, fixed in Davidson’s solution and embedded in paraffin. Then, 5-mm thick sections from each tissue were microdissected and stained with Harris’ haematoxylin and eosin and examined using a light microscope for histopathological analysis. HN was diagnosed and classified according to three disease stages (i.e. N1, N2, or N3) as follows. N1 stage: small groups of leukaemic cells were detected only in the vessels of the gills and in the connective tissue surrounding the digestive tubules. N2 stage: leukaemic cells spread to different organs, conforming small groups in the connective tissue that surrounds the digestive gland and the gonadal follicles, branchial sinuses, and mantle. N3 stage: leukaemic cells invade the filaments, completely deforming the plica structure in the gill, invade the connective tissue surrounding the gonadal follicles and the digestive gland; in the mantle, they invade the connective tissue, but in the muscle fibres of the mantle and foot, cells appear isolated or in small groups and in lower intensity than in other tissues.

### Electron microscopy analysis

Four *V. verrucosa* specimens (two non-neoplastic, ERVV17-2993 and ERVV17-2992, and two with high grade of HN, ERVV17-2995 and ERVV17-3193) were processed for transmission electron microscopy as follows: 2 mm sections of gills and digestive glands were fixed in 2.5% glutaraldehyde seawater for 2 hr at 4°C. Then, tissues were post-fixed in 1% osmium tetroxide in sodium cacodylate solution and embedded in Epon resin. Ultrathin sections were stained with uranyl acetate and lead citrate and examined in a JEM-1010 transmission electron microscope.

### Cytogenetics

Mitotic chromosomes of a neoplastic *V. verrucosa* specimen (EVVV11-02) were obtained as follows. After colchicine treatment (0.005%, 10 hr), gills were dissected, treated with a hypotonic solution, and fixed with ethanol and acetic acid. Small pieces of fixed gills were disaggregated with 60% acetic acid to obtain cell suspensions that were spread onto preheated slides. Chromosome preparations were stained with DAPI (0.14 mg/ml) and PI (0.07 mg/ml) for 8 min, mounted with antifade medium, and photographed. A comparative screening of tandem repeats was performed on the genomes of *C. gallina* and *V. verrucosa* using RepeatExplorer (Novák et al., 2010) on a merged short-read dataset of both species (500,000 reads each). Short reads of healthy and neoplastic animals were mapped onto both satellite consensus sequences using BWA, filtered according to their mapping quality (q > 60 and AS >70) and their abundance assessed by means of samtools/bamtools. Satellites CL4 and CL17 were selected for FISH purposes and FISH probes were PCR amplified (CL4F: TCAGAAACCGCTATTTTTCAC, CL4R: AAATGATGCTACGAACCTCC and CL17F: ATTCCAGAAATGTACATGAACAC, CL17R: ATTTTTGCACCAGATGTTCAC, respectively) and directly labelled with digoxigenin-11-dUTP (10× DIG Labeling Mix, Roche Applied Science). FISH experiments were performed as described in reference (García-Souto et al., 2015).

### De novo assembly of mitochondrial genomes and annotation

In total, we performed whole-genome sequencing on 23 samples from 16 clam specimens, which includes 8 neoplastic and 8 non-neoplastic animals by Illumina paired-end libraries of 350 bp insert size and reads 150 bp long. First we assembled the mitochondrial genomes of one *V. verrucosa* (FGVV18_193), one *C. gallina* (ECCG15_201), and one *C. striatula* (EVCS14_02) specimens with MITObim v1.9.1 (Hahn et al., 2013), using gene baits from the following *Cox1* and *16*S reference genes to prime the assembly of clam mitochondrial genomes: *V. verrucosa* (*Cox1*, with GenBank accession number KC429139; and *16*S: C429301), *C. gallina* (*Cox1*: KY547757, *16*S: KY547777), and *C. striatula* (*Cox1*: KY547747, *16*S: KY547767). These draft sequences were polished twice with Pilon v1.23 (Walker et al., 2014), and conflictive repetitive fragments from the mitochondrial control region were resolved using long read sequencing with Oxford Nanopore technologies (ONT) on a set of representative samples from each species and tumours. ONT reads were assembled with Miniasm v0.3 (Li, 2016) and corrected using Racon v1.3.1 (Vaser et al., 2017). Protein-coding genes, rDNAs and tDNAs were annotated on the curated mitochondrial genomes using MITOS2 web server (Bernt et al., 2013), and manually curated to fit ORFs as predicted by ORF-FINDER (Rombel et al., 2002). Then, we employed the entire mtDNAs of *V. verrucosa* (FGVV18_193) and *C. gallina* (ECCG15_201) as ‘references’ to map reads from individuals with neoplasia, filter reads matching either mitogenome and assemble and polish their two (healthy and tumoural) mitogenomes individually as above. Further healthy individuals were later sequenced and their mitogenomes assembled, to further investigate the geographic and taxonomic spread of this neoplasia.

### Analysis of *Cox1* sequences

We retrieved a dataset of 3745 sequences comprising all the barcode-identified venerid clam *Cox1* fragments available from the Barcode of Life Data System (BOLD, http://www.boldsystemns.org/). Redundancy was removed using CD-HIT (Fu et al., 2012), applying a cut-off of 0.9 sequence identity, and sequences were trimmed to cover the same region. Whole-genome sequencing data from both healthy and tumoural warty venus clams were mapped onto this dataset, containing 118 venerid species-unique sequences, using BWA-mem, filtering out reads with mapping quality below 60 (-q60), and quantifying the overall coverage for each sequence with samtools idxstats. PCR primers were designed with Primer3 v2.3.7 (Kõressaar et al., 2018) to amplify a fragment of 354 bp from the *Cox1* mitochondrial gene of *V. verrucosa* and *C. gallina* (F: CCT ATA ATA ATT GGK GGA TTT GG, R: CCT ATA ATA ATT GGK GGA TTT GG). PCR products were purified with ExoSAP-IT and sequenced by Sanger sequencing.

### Mitochondrial genome coverage analysis

We further mapped the paired-end sequencing data from healthy and neoplastic tissues from all neoplastic samples onto the ‘reference’ mitochondrial genomes of *V. verrucosa* and *C. gallina* (two of the previously assembled ones, FGVV18_193 and ECCG15_201) using BWA-mem v0.7.17-r1188 (Li and Durbin, 2009) with default parameters. Duplicate reads were marked with Picard 2.18.14 and removed from the analysis. Read coverage depth was computed with samtools v1.9 (Li et al., 2009), summarized by computing the average in windows of 100 bp size and plotted with R v3.5.3.

### Draft assembly of nuclear reference genomes, identification of variable single copy orthologous nuclear loci and SNPs

We ran the MEGAHIT v1.1.3 assembler (Li et al., 2015) on the Illumina paired-end sequencing data to obtain partial nuclear genome assemblies of *V. verrucosa* (FGVV18_193), *C. gallina* (ECCG15_201), and *C. striatula* (EVCS14_02). Then, single copy genes were predicted with Busco v.3.0.2 (Seppey et al., 2019). Candidate genes were considered if they (1) were present in the genomes of the three species, and (2) showed variant allele frequencies (VAFs) at exclusively 0, 0.5, or 1.0 in all the sequenced healthy (non-neoplastic) specimens. Under this criteria, two loci were finally selected: a 3914-bp long fragment of *DEAH12*, a gene encoding for an ATP-dependent RNA helicase and a 2.2-kp length fragment of the Transcription Factor II Human-like gene, *TFIIH*. PCR primers were designed with Primer3 v2.3.7 to amplify and sequence a 441-bp region of the *DEAH12* nuclear gene (DEAH12_F: AGGTATGCTGAAACAAACACTT and DEAH12_R: ACGACAAATTTGATACCTGGAAT) and a 559-bp fragment of the *TFIIH* gene (TFIIH_F: TGGCATCTTTGTTACGGAC and TFIIH_R: CTTGTGRTTCTGTATCTGATCAATAA) on neoplastic specimens from *V. verrucosa* and healthy animals from both species (*DEAH12*: 11 *V*. *verrucosa* and 9 *C. gallina*; *TFIIH*: 15 *V*. *verrucosa* and 12 *C*. *gallina*). We screened for differentially fixed SNVs between both species using the dapc function in the R package Exploratory Analysis of Genetic and Genomic Data adegenet (Jombart and Ahmed, 2011). These variants were later employed to filter the Illumina short reads matching either *V. verrucosa* or *C. gallina* genotypes from the neoplastic animals, and to obtain consensus sequences from tumour and healthy tissue in each sequenced specimen. Read filtering was performed with samtools fillmd, while GATK mutect2 (Benjamin et al., 2019) was used for variant calling. Only variants with VAFs close to fixation (>0.9) were considered when building the consensus sequences.

### Phylogenetic analyses

Mitochondrial sequences for 13 coding genes and 2 rDNA genes from the 23 recovered mitogenomes (6 neoplastic, 17 from host and healthy specimens) were extracted from the paired-end sequencing data by mapping reads onto the previously reconstructed canonical mtDNAs for *V. verrucosa* and *C. gallina* (Figure 2—figure supplement 1), concatenated, and subjected to multiple alignment with MUSCLE v3.8.425 (Edgar, 2004). The best-fit model of nucleotide substitution for each individual gene was selected using JModelTest2 (Darriba et al., 2012) and a partitioned Bayesian reconstruction of the phylogeny was performed with MrBayes v3.2.6 (Ronquist et al., 2012). Two independent Metropolis-coupled Markov Chain Monte Carlo (MCMC) analyses with four chains in each were performed. Each chain was run for 10 million generations, sampling trees every 1000 generations. Convergence of runs was assessed using Tracer (Rambaut et al., 2018).

*DEAH12* and *TFIIH* sequences were subjected to multiple alignment using MUSCLE v3.8.425. Then, a ‘species/population tree’ was inferred with the starBEAST multispecies coalescent model, as implemented in BEAST v2.6.2 (Bouckaert et al., 2019). This analysis was performed using a Yule speciation prior and strict clock, with the best-fit model of nucleotide substitution obtained with jModelTest2 on both the concatenated mitochondrial haplotypes (13 protein-coding and 2 rRNAs genes) and unphased data from *DEAH12* and *TFIIH* nuclear fragments. The four mitochondrial groups observed on the mitogenome analysis (*V. verrucosa*, *C. gallina*, *C. striatula*, and Tumour) were defined as tips for the species tree. A single MCMC of 10 million iterations, with sampling every 1000 steps, was run. A burn-in of 10% was implemented to obtain ESS values above 200 with Tracer v1.7.1 and the resulting posterior distributions of trees were checked with DENSITREE v2.1 (Bouckaert, 2010). A maximum clade credibility tree was obtained with TreeAnnotator (Bouckaert et al., 2019) to summarize information on topology, with 10% burn-in and Common Ancestors for the node heights.

## Data Availability

Nucleotide data for the mitochondrial DNA assemblies has been uploaded to GenBank under accession numbers MW662590-MW662611. These correspond to reference healthy animals of Venus verrucosa (MW662590, MW662593, MW662607, MW662608 and MW662610), Chamelea gallina (MW662591 and MW662609), Chamelea striatula (MW662611) and to both normal (MW662592, MW662595, MW662597, MW662599, MW662601, MW662602, MW662604, MW662606) and tumoral (MW662594, MW662596, MW662598, MW662600, MW662603, MW662605) mitochondrial DNAs of neoplastic animals. Genomic data (Illumina paired-end sequencing) from the mitogenomes analysed in this manuscript are allocated in DRYAD (https://doi.org/10.5061/dryad.zcrjdfn9v). The following dataset was generated: Garcia-SoutoD Diaz-CostasS BruzosA RochaS Roman-LewisC AlonsoJ RodriguezR JorgeR VillanuevaA SilvaL ValenciaJ AnnonaG TaralloA RicardoF Bratos-CetinicA PosadaD PasantesJ Tubio JMC 2021Mitochondrial genome sequencing of marine leukemias reveals cancer contagion between clam species in the Seas of Southern EuropeDryad Digital Repository10.5061/dryad.zcrjdfn9vPMC876575235040778 Garcia-SoutoD Diaz-CostasS BruzoAL RochaS PequenoA Roman-LewisCF CostasD Rodriguez-CastroJ VillanuevaA SilvaL ValenciaJM AnnonaG TaralloA RichardoF Bratos-CentinicA PosadaD TubioJM NCBI GenBank2022Venus verrucosa mitochondrion, complete genome CSVV18_1050 Healthy animal, footMW662590 Garcia-SoutoD Diaz-CostasS BruzosAL RochaS PequenoA Roman-LewisCF CostasD Rodriguez-CastroJ VillanuevaA SilvaL ValenciaJM AnnonaG TaralloA RicardoF Bratos-CetinicA TubioJM NCBI GenBank2022Venus verrucosa mitochondrion, complete genome EMVV18_385 Healthy animal, footMW662593 Garcia-SoutoD Diaz-CostasS BruzosAL RochaS PequenoA Roman-LewisCF CostasD Rodriguez-CastroJ VillanuevaA SilvaL ValenciaJM TaralloA RichardoF Bratos-CetinicA PosadaD TubioJM NCBI GenBank2022Venus verrucosa mitochondrion, complete genome FGVV18_193 Healthy animal, footMW662607 Garcia-SoutoD Diaz-CostasS BruzoAL RochaS PequenoA Roman-LewisCF CostasD Rodriguez-CastroJ VillanuevaA SilvaL ValenciaJM AnnonaG TaralloA RichardoF Bratos-CetinicA PosadaD TubioJM NCBI GenBank2022Venus verrucosa mitochondrion, complete genome IGVV19_666 Healthy animal, footMW662608 Garcia-SoutoD Diaz-CostasS BruzosAL RochaS PequenoA Roman-LewisCF CostasD Rodriguez-CastroJ VillanuevaA SilvaL ValenciaJM AnnonaG TaralloA RichardoF Bratos-CetinicA PosadaD TubioJM NCBI GenBank2022Venus verrucosa mitochondrion, complete genome PLVV18_2249 Healthy animal, footMW662610 Garcia-SoutoD Diaz-CostasS BruzosAL RochaS PequenoA Roman-LewisCF CostasD Rodriguez-CastroJ VillanuevaA SilvaL ValenciaJM AnnonaG TaralloA RichardoF Bratos-CetinicA PosadaD TubioJM NCBI GenBank2022Chamelea gallina mitochondrion, complete genome ECCG15_201 Healthy animal, footMW662591 Garcia-SoutoD Diaz-CostasS BruzoAL RochaAL PequenoA Roman-LewisCF CostasD Rodriguez-CastroJ VillanuevaA SilvaL ValenciaJM AnnonaG TaralloA RichardoF Bratos-CetinicA PosadaD TubioJM NCBI GenBank2022Venus verrucosa mitochondrion, complete genome EMVV18_376 Normal mitochondria from neoplastic animal, footMW662592 Garcia-SoutoD Diaz-CostasS BruzosAL RochaS PequenoA Roman-LewisCF CostasD Rodriguez-CastroJ VillanuevaA SilvaL ValenciaJM AnnonaG TaralloA RichardoF Bratos-CetinicA PosadaD TubioJM NCBI GenBank2022Venus verrucosa mitochondrion, complete genome EMVV18_391_Normal Normal mitochondria from neoplastic animal, footMW662595 Garcia-SoutoD Diaz-CostasS BruzoAL RochaA Roman-LewisCF CostasD Rodriguez-CastroJ VillanuevaA SilvaL ValenciaJM AnnonaG TaralloA RichardoF Bratos-CetinicA PosadaD TubioJM PequenoA NCBI GenBank2022Venus verrucosa mitochondrion, complete genome EMVV18_395_Normal Normal mitochondria from neoplastic animal, footMW662597 Garcia-SoutoD Diaz-CostasS BruzosAL RochaS PequenoA Roman-LewisCF CostasD Rodriguez-CastroJ VillanuevaA SilvaL ValenciaJM AnnonaG TaralloA RichardoF Bratos-CetinicA PosadaD TubioJM NCBI GenBank2022Venus verrucosa mitochondrion, complete genome EMVV18_400_Normal Normal mitochondria from neoplastic animal, footMW662599 Garcia-SoutoD Diaz-CostasS BruzosAL RochaS PequenoA Roman-LewisCF CostasD Rodriguez-CastroJ VillanuevaA SilvaL ValenciaJM AnnonaG TaralloA RichardoF Bratoc-CetinicA PosadaD TubioJM NCBI GenBank2022Venus verrucosa mitochondrion, complete genome ERVV17_2995_Normal Normal mitochondria from neoplastic animal, footMW662601 Garcia-SoutoD Diaz-CostasS BruzosAL RochaS PequenoA Roman-LewisCF CostasD Rodriguez-CastroJ VillanuevaA SilvaL ValenciaJM AnnonaG TaralloA RichardoF Bratos-CetinicA PosadaD TubioJM NCBI GenBank2022Venus verrucosa mitochondrion, complete genome ERVV17_2997_Normal Normal mitochondria from neoplastic animal, footMW662602 Garcia-SoutoD Diaz-CostasS BruzosAL RochaS PequenoA Roman-LewisCF CostasD Rodriguez-CastroJ VillanuevaA SilvaL ValenciaJM AnnonaG TaralloA RichardoF Bratos-CetinicA PosadaD TubioJM NCBI GenBank2022Venus verrucosa mitochondrion, complete genome ERVV17_3193_Normal Normal mitochondria from neoplastic animal, footMW662604 Garcia-SoutoD Diaz-CostasS BruzosAL RochaS PequenoA Roman-LewisCF CostasD Rodriguez-CastroJ VillanuevaA SilvaL AnnonaG TaralloA RichardoF Bratos-CetinicA PosadaD TubioJM ValenciaJM NCBI GenBank2022Venus verrucosa mitochondrion, complete genome EVVV11_2_Normal Normal mitochondria from neoplastic animal, footMW662606 Garcia-SoutoD Diaz-CostasS BruzosAL RochaS PequenoA Roman-LewisCF CostasD Rodriguez-CastroJ VillanuevaA SilvaL ValenciaJM AnnonaG TaralloA RichardoF Bratos-CetinicA PosadaD TubioJM GenBank2022Venus verrucosa mitochondrion, complete genome EMVV18_391_Tumour Transmissible neoplasia derived from Chamelea gallina, neoplastic hemocytesMW662594 Garcia-SoutoD Diaz-CostasS BruzosAL RochaS PequenoA Roman-LewisCF CostasD Rodriguez-CastroJ VillanuevaA SilvaL ValenciaJM AnnonaG TaralloA RichardoF Bratos-CetinicA PosadaD TubioJM NCBI GenBank2022Venus verrucosa mitochondrion, complete genome EMVV18_395_Tumour Transmissible neoplasia derived from Chamelea gallina, neoplastic hemocytesMW662596 Garcia-SoutoD Diaz-CostasS BruzosAL RochaS PequenoA Roman-LewisCF CostasD Rodriguez-CastroJ VillanuevaA SilvaL ValenciaJM AnnonaG TaralloA RichardoF Bratos-CetinicA PosadaD TubioJM NCBI GenBank2022Venus verrucosa mitochondrion, complete genome EMVV18_400_Tumour Transmissible neoplasia derived from Chamelea gallina, neoplastic hemocytesMW662598 Garcia-SoutoD Diaz-CostasS BruzosAL RochaS PequenoA Roman-LewisCF CostasD Rodriguez-CastroJ VillanuevaA SilvaL ValenciaJM AnnonaG TaralloA RichardoF Bratos-CetinicA PosadaD TubioJM NCBI GenBank2022Venus verrucosa mitochondrion, complete genome ERVV17_2995_Tumour Transmissible neoplasia derived from Chamelea gallina, neoplastic hemocytesMW662600 Garcia-SoutoD Diaz-CostasS BruzosAL RochaS PequenoA Roman-LewisCF CostasD Rodrigues-CastroJ VillanuevaA SilvaL ValenciaJM AnnonaG TaralloA RicardoF Bratos-CetinicA PosadoD TubioJM NCBI GenBank2022Venus verrucosa mitochondrion, complete genome ERVV17_3193_Tumour Transmissible neoplasia derived from Chamelea gallina, neoplastic hemocytesMW662603 Garcia-SoutoD Diaz-CostaS BruzosAL RochaS PequenoA Roman-LewisCF CostasD Rodriguez-CastroJ VillanuevaA SilvaL ValenciaJM AnnonaG TaralloA RichardoF Bratos-CetinicA PosadaD TubioJM NCBI GenBank2022Venus verrucosa mitochondrion, complete genome EVVV11_2_Tumour Transmissible neoplasia derived from Chamelea gallina, neoplastic hemocytesMW662605 Garcia-SoutoD Diaz-CostasS BruzoAL RochaS PequenoA Roman-LewisCF CostasD Rodriguez-CastroJ VillanuevaA SilvaL ValenciaJM AnnonaG TaralloA RichardoF Bratos-CetinicA PosadaD TubioJM 2022Chamelea gallina mitochondrion, complete genome IMCG15_69 Healthy animal, footNCBI GenBankMW662609 Garcia-SoutoD Diaz-CostasS BruzosAL RochaS PequenoA Roman-LewisCF CostasD Rodriguez-CastroJ VillanuevaA SilvaL ValenciaJM AnnonaG TaralloA RichardoF Bratos-CetinicA PosadaD TubioJM 2022Chamelea striatula mitochondrion, complete genome EVCS14_09 Healthy animal, footNCBI GenBankMW662611

## Additional files

Supplementary file 1. Sampling data of 570 specimens analysed in this study.

Transparent reporting form
